# Draft Genome Sequence of the Immunobiotic Strain *Lactobacillus jensenii* TL2937

**DOI:** 10.1128/genomeA.00005-17

**Published:** 2017-03-02

**Authors:** Julio Villena, Yuki Masumizu, Hikaru Iida, Wakako Ikeda-Ohtsubo, Leonardo Albarracin, Seiya Makino, Sou Ohkawara, Katsunori Kimura, Lucila Saavedra, Elvira Maria Hebert, Haruki Kitazawa

**Affiliations:** a Reference Centre for Lactobacilli (CERELA-CONICET), San Miguel de Tucumán, Tucumán, Argentina; b Food and Feed Immunology Group, Laboratory of Animal Products Chemistry, Graduate School of Agricultural Science, Tohoku University, Sendai, Japan; c Livestock Immunology Unit, International Education and Research Center for Food Agricultural Immunology (CFAI), Graduate School of Agricultural Science, Tohoku University, Sendai, Japan; d Food Science Research Laboratories, R&D Division, Meiji Co., Ltd., Kanagawa, Japan; e Agricultural and Veterinary Division, Meiji Seika Pharma Co., Ltd., Tokyo, Japan

## Abstract

The genome of the immunomodulatory strain *Lactobacillus jensenii* TL2937 is described here. The draft genome has a total length of 1,678,416 bp, a G+C content of 34.3%, and 1,470 predicted protein-coding sequences. The genome information will be useful for gaining insight into the immunomodulatory properties of the TL2937 strain in the porcine host.

## GENOME ANNOUNCEMENT

Probiotics able to modulate the immune system (immunobiotics) are of value for reducing intestinal inflammation in pigs (1). *Lactobacillus jensenii* TL2937 inhibits nuclear factor κB and mitogen-activated protein kinase signaling pathways in porcine intestinal epithelial (PIE) cells after the activation of TLR4 through an upregulation of the negative regulators MKP-1, A20, and Bcl-3, conferring protection against inflammatory damage (2). Microarray analysis indicated that *L. jensenii* TL2937 stimulation decreased the expression of cytokines, chemokines, and adhesion molecules in PIE cells (3). *L. jensenii* TL2937 is also able to modulate antigen-presenting cells (APCs) from porcine Peyer’s patches (PPs) and blood (4, 5). Stimulation of PPs and blood porcine APCs with the TL2937 strain resulted in a differential cytokine profile in response to TLR4 activation (4). This effect was partially dependent on TLR2 activation and completely dependent on efficient phagocytosis (5). Our *in vivo* experiments in pigs demonstrated that the administration of TL2937 improved immune health, growing performance, and productivity of piglets (6).

The genome of *L. jensenii* TL2937 was sequenced using a whole-genome shotgun strategy on an Illumina MiSeq sequencer. Paired reads with lengths of 300 bp were obtained corresponding to a 1,267-fold coverage. Quality-filtered reads were assembled using Ngen version 12.2.0 software (DNASTAR). This genome was assembled into 69 contigs (mean coverage of 1,267.0×). The functional annotation of predicted genes in the *L. jensenii* TL2937 genome was achieved using the RAST server and the NCBI’s Prokaryotic Genome Annotation Pipeline (7). tRNAs and rRNAs were identified by tRNAscan-SE and RNAmmer, respectively (8, 9).

The draft genome of *L. jensenii* TL2937 consists of 1,678,416 bp with a mean G+C content of 34.3%. A total of 1,470 coding sequences (CDSs), 53 structural tRNAs, and nine rRNAs were predicted. Among all CDSs, 1,157 (70%) were assigned to known protein functions, while the remaining 313 (30%) were identified as hypothetical proteins. Additionally, there are 251 RAST subsystems represented in the genome, which represent only 47% of the assigned sequences. Interestingly, *in silico* genomic studies revealed an open reading frame encoding a putative fibronectin-binding protein (BFX48_RS04115) consisting of 563 amino acids that appears to be highly conserved among *Lactobacillus* species (10). Fibronectin-binding proteins are extensively described in bacterial pathogens; however, it was postulated that in probiotic strains these adhesion molecules are essential for attachment to their ecological niches (10). Buck et al. (11) reported that a *L. acidophilus* mutant, with inactivated fbpA, exhibited a significant decrease in adhesion to epithelial cells *in vitro* (11). Based on these data, ongoing research is focused on the functional characterization of this putative adhesive molecule.

The draft genome sequence of *L. jensenii* TL2937 will be useful for further studies of specific genetic features of this strain, for understanding the mechanisms of its immunobiotic properties in the porcine host, and for its biotechnological application in the development of novel functional feeds.

### Accession number(s).

This whole-genome shotgun project has been deposited at DDBJ/EMBL/GenBank under the accession number MDTN01000000. The version described in this paper is the first version, MDTN01000000.1.
